# Temperature dependent transitions in excitability predicted by an electrodiffusion model of membrane potential

**DOI:** 10.1186/1471-2202-13-S1-P85

**Published:** 2012-07-16

**Authors:** Juan R Melendez-Alvarez, Erin C McKiernan, Marco Arieli Herrera Valdez

**Affiliations:** 1 Department of Mathematics and Physics, University of Puerto Rico at Cayey, Cayey, PR, 00736, USA; 2 Institute of Interdisciplinary Research, University of Puerto Rico at Cayey, Cayey, PR, 00736, USA

## 

Temperature affects cellular function in different ways that include the transmembrane transport of ions and the kinetics of membrane spanning proteins mediating such transport. Electrodiffusion models of membrane potential [1,2]can be used to study the temperature dependence of the kinetics of the channels and the transmembrane diffusion of ions. In turn, channel kinetics and transmembrane diffusion may cause significant changes in the dynamics of a model neuronal membrane. We constructed a two-dimensional biophysical electrodiffusion model to study the specific effects on the excitability profiles of the model neuron as a function of temperature. The formulations of the model allows an analysis that can be interpreted in terms of patterns of ion channel expression [3,4]. A change in the variables of the system unravels the model to be rewritten so that only one of the variables is temperature-dependent. We use bifurcation analysis to map the possible excitability profiles of the model neuron . To do so, we use an external current stimulus as a bifurcation parameter, for a range of temperatures and for different patterns of ion channel expression. The bifurcation structure of the model is used to generate basic intuition and explanations for the respose profiles of the model neuron subject to stimuli that includes square pulses, ramps, and synaptic input. We identify parameter regimes associated with specific patterns of ion channel expression in which the excitability of the membrane undergoes significant changes. We identify possible compensation mechanisms not requiring enzymatic actions that may underlie the regularization of cellular function within the nervous systems of animals exposed to different temperatures.
